# The TTCYB Study Protocol: A Tailored Print Message Intervention to Improve Cardiovascular Patients’ Lifestyles

**DOI:** 10.3390/ijerph17082919

**Published:** 2020-04-23

**Authors:** Marco D’Addario, Erika Rosa Cappelletti, Marcello Sarini, Andrea Greco, Patrizia Steca

**Affiliations:** 1 Department of Psychology, University of Milano-Bicocca, 20126 Milan, Italy; marcello.sarini@unimib.it (M.S.); patrizia.steca@unimib.it (P.S.); 2 Health Promotion Division, Agenzia Tutela Salute Milano, 20129 Milan, Italy; ercappelletti@ats-milano.it; 3 Department of Human and Social Sciences, University of Bergamo, 24129 Bergamo, Italy; andrea.greco@unibg.it

**Keywords:** tailored communication, chronic disease, self-management and lifestyle behaviors, cardiovascular diseases

## Abstract

This article describes the development of the “Time to Change Your Behavior” (TTCYB) study protocol, a theory-based, tailored print message intervention to improve compliance with the self-care regimen in patients with cardiovascular diseases. A design with a baseline measurement and two follow-ups at six and 12 months will be applied. At baseline and the six-month follow-up, patients will complete self-report questionnaires evaluating lifestyle habits and socio-demographic and psychological variables; at the 12-month follow-up, patients will answer a telephone interview assessing lifestyle habits. After the baseline measurement, patients will be randomized into one of three groups: (1) the tailored group, which will receive tailored health brochures; (2) the “non-tailored” group, which will receive non-tailored health brochures; or (3) the usual care group, which will receive no print information materials. The effectiveness of the intervention will be assessed through patients’ judgments of the brochures and changes in lifestyle. The role of socio-demographic and psychological variables as potential moderators of the materials’ effectiveness will be explored. If the TTCYB is efficacious, it will have implications for the design and implementation of tailored communication programs. Concepts from this study can be potentially extended to primary prevention among high-risk groups.

## 1. Introduction

Mortality statistics show that cardiovascular diseases (CVDs) are the most common causes of death in Europe, accounting for 45% of all deaths. More than four million people die from CVDs across Europe every year, with 1.4 million of these deaths before the age of 75 [1]. Several guidelines for the detection and management of CVDs consider lifestyle behavior change a best practice for avoiding poor outcomes related to disease severity and concurrent comorbid risk factors [2,3,4,5,6], but patients often fail to change their unhealthy habits [7,8,9]. Education about lifestyle change is a priority for patients’ health [10], and strategically designed and delivered health communication interventions offer promising strategies for promoting healthier behaviors. One of the most challenging issues in health promotion research is the creation and delivery of messages that are relevant, interesting, informative, and persuasive. Tailored print communication based on the needs, preferences, and personal characteristics of the target is an example of a communication approach that has attempted to address these characteristics and has been widely applied in the area of health promotion [11,12,13]. Tailored messages are thought to facilitate changes in several behaviors by providing personally relevant information and feedback [11]. Meta-analyses and narrative reviews have concluded that tailored messages are more effective than non-tailored ones in changing health behavior in multiple domains [13,14,15,16,17,18,19,20], including medication adherence [21], physical activity [22], and diet [23,24], all key aspects for the management of cardiovascular diseases. To date, little is known about the efficacy and the long-term outcomes of tailored versus non-tailored communication interventions: a large majority of studies on tailored interventions focused on short-term effects of tailored messages while few studies investigated long-term effects (most of them analyzed results up to six months after the intervention). Mobile technology is changing this perspective because the use of technology makes longitudinal studies more practically feasible, even if some subjects’ characteristics (e.g., age, digital skills, web accessibility) can limit their realization [14,25]. The importance of a healthy lifestyle for positive health outcomes coupled with the low reported rate of correct behaviors by cardiovascular patients prompts research to define communication strategies to improve adherence in this population. Much research is needed to determine the degree of the effectiveness of tailored communication interventions among cardiovascular patients.

### The “Time to Change Your Behavior” (TTCYB) Intervention

The “Time to Change Your Behavior” (TTCYB) is a randomized controlled intervention that uses tailored health print educational material to improve healthy lifestyle behaviors (dietary behavior, physical activity, alcohol intake and smoking behavior) in patients affected by acute coronary syndrome (ACS) or essential hypertension. It is a pragmatic intervention that primes the patient to self-manage by making relevant changes in the following four behaviors: diet, physical activity, alcohol intake, and smoking. The purpose of this paper is to outline the rationale and design of the TTCYB Intervention Study. This study will provide insight into the effect of tailored print educational materials in promoting lifestyle changes among patients diagnosed with CVDs. The specific aims of the study will be

- to determine whether tailored materials are perceived as more useful, understandable, and complete compared to non-tailored ones;
- to understand whether the TTCYB intervention is effective in promoting changes in diet, alcohol intake, physical activity, and smoking behavior among patients with CVDs;
- to explore the effects of the TTCYB intervention compared with two control groups (described below) on secondary endpoints, including body mass index and systolic and diastolic blood pressure;
- to evaluate socio-demographic and psychological factors such as self-efficacy, locus of control, and anxiety and depression, which could moderate the effectiveness of the intervention.

The choice to develop an intervention using health print educational material comes from different reasons. Given the global scale of CVDs, preventive interventions that can reach large populations at low cost are needed. Mobile app or computer-delivered interventions have become increasingly popular in the last decade. However, tailored interventions delivered on the Web or smartphones can be especially challenging among specifics segments of the population, like older adults. Moreover, data on internet use show that a digital divide still exists around the world and Italy lags behind other European countries. A recent study has compared general social media use in the United Kingdom, Germany, the Netherlands, and Italy, showing that the latter makes the least use of them, with 40% (37 million) of the Italian population active [26]. Moreover, patients affected by acute coronary syndrome and hypertension seemed to prefer and considered more relevant print health material given by physicians compared to the internet [27]. Thus, because offering interventions on the Web or through mobile applications may exclude a vulnerable group, we preferred to use health print educational material. 

## 2. Materials and Methods 

### 2.1. Design

**Figure 1:**
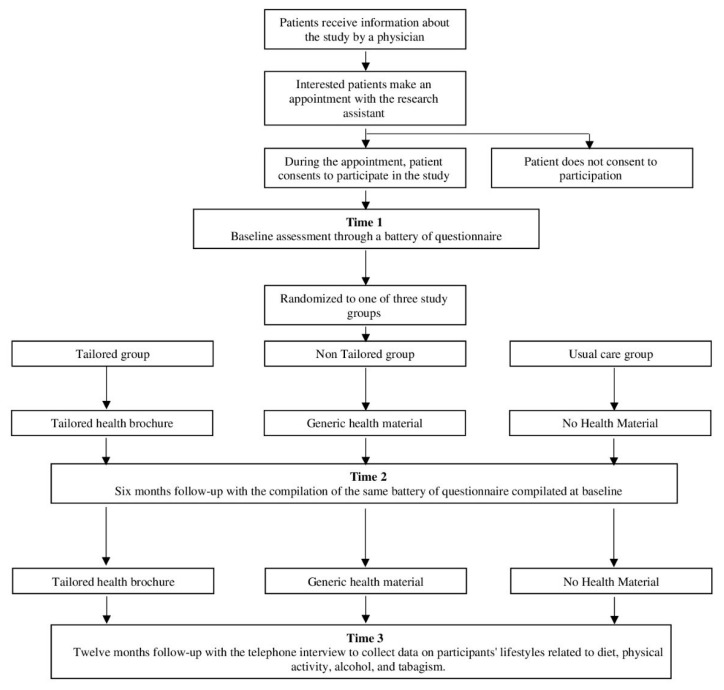
Flowchart of study participation.

The TTCYB study is a three-arm, parallel-group, randomized intervention designed to determine the effect of tailored print materials for patients with CVDs (see Figure 1). Follow-up assessments will occur at six and 12 months post-baseline evaluation.

At baseline and the six-month follow-up, patients will complete a series of self-report questionnaires evaluating lifestyle behavior, socio-demographic, and psychological characteristics. At the 12-month follow-up, patients will have a telephone interview that will assess lifestyle habits. After the baseline measurement, patients will be randomized into one of three groups: (1) the tailored group, which will receive tailored health brochures; (2) the “non-tailored” group, which will receive non-tailored health brochures; and (3) the usual care group, which will receive no print information materials. Participants will be assigned to one of the three study groups using a stratified randomization process characterized by the unpredictability of assignments. Patients in the tailored and non-tailored group will be mailed a health brochure after a baseline and a six-month evaluation. Within ten working days of completing the questionnaire, both at baseline and the six-month follow-up, the tailored (T) and non-tailored (NT) group will be mailed printed health materials while patients in the usual care (UC) group will receive no materials. Following the delivery of the materials, patients in the T and NT group will be contacted by phone for an interview to measure their judgment of the material. At the 12-month follow-up, all patients will have a telephone interview that will assess their lifestyle in terms of dietary behavior, physical activity, alcohol intake, and smoking behavior.

### 2.2. Recruitment of Participants

Patients attending their specialist examination appointments will be advised about the study by physicians. Physicians will identify potentially eligible patients and will initiate discussion of the study with them. After discussion with physicians, interested patients will be asked to book an appointment on one of the pre-determined recruitment days to speak to a research assistant about the study. During the scheduled appointment, the research assistant will explain the different phases of the study and will obtain informed consent from patients who will agree to participate.

### 2.3. Eligibility

All participants will have to meet the following eligibility criteria: 18 years of age or older; diagnosis of essential arterial hypertension (SBP ≥ 140 mmHg and/or DBP ≥ 90 mmHg, evaluated in the standard way or by 24-h mean arterial pressure (MAP) value monitoring) or ACS; able to complete the study measures and interventions in Italian; without other serious diseases (such as cancer) or psychiatric disorders that could compromise the participation in the study.

### 2.4. Measures

For an overview of all measures over the different measurement time points, please see Table 1. The background variables that will be assessed both at baseline and at the six-month follow-up are explained in the following paragraphs. 

#### 2.4.1. Dietary Behavior

To measure dietary behavior, a modified version of the Mediterranean Diet Scale (MDS), [28] will be used. The instrument measures the weekly consumption of nine foods using a six-point Likert scale, where 1 indicates “Never” and 6 indicates “More than three times a day”. The consumption of both beneficial (i.e., vegetables, fruits, whole grains, fish, legumes, olive oil, up to two glasses of wine per day) and detrimental foods (i.e., more than two glasses of wine per day, butter and margarine, red or processed meat) will be assessed. Each response is recoded into a dichotomous variable according to Trichopoulou et al. [28], where 1 indicates healthy and 0 indicates unhealthy consumption. The sum of the recoded responses yields the Mediterranean Diet Scale (MDS) score, on which higher scores indicate a healthier diet. 

#### 2.4.2. Physical Activity

Physical activity will be measured with the Rapid Assessment of Physical Activity-1 (RAPA-1) [29], a self-report instrument to assess physical activity in clinical contexts [30,31,32]. The RAPA consists of seven hierarchical questions about different intensity levels and frequency of physical activity and aerobic exercise. The intensity levels of the activity (light, moderate, vigorous) are described and examples of them are given. Each question requires a dichotomous (yes/no) response, where “yes” is scored 1 and “no” is scored 0. The total score is obtained by adding the scores of individual items and it ranges from 1 (“I rarely or never do any physical activity”) to 7 (“I do 20 min or more of vigorous physical activity a day, three or more days a week”). 

#### 2.4.3. Alcohol Intake

The total alcohol intake for each participant will be computed as the mean of the contribution from beer (1 = “I don’t drink beer”, 2 = “Up to two glasses a day”, 3 = “three or four glasses a day”, 4 = “More than four glasses a day”), wine (1 = “I don’t drink wine”, 2 = “Up to 2 glasses a day”, 3 = “Three or four glasses a day”, 4 = “More than four glasses a day”), and spirits (1 = “I don’t drink spirits”, 2 = “One glass occasionally—a few times a year”, 3 = “one glass habitually—e.g., every week, always after meals, etc.”, 4 = “More than one glass habitually—e.g., every week, always after meals etc.”), as used in a previous research [33,34]. 

#### 2.4.4. Smoking Behavior

Smoking behavior will be measured by the Fagerström Test for Nicotine Dependence [35], a six-item scale that evaluates the quantity of cigarette consumption, the compulsion to use, and dependence. The higher the total Fagerström score is, the more intense is the patient’s dependence on nicotine. 

#### 2.4.5. HAPA Constructs

The intervention will be guided by Schwarzer’s Health Action Process Approach (HAPA) model [36,37]. The questions used to measure the HAPA constructs have been developed following suggestions from the authors of the model [36,37].

Risk perception will be assessed using three questions on a 5-point Likert scale, where 1 indicates “Not at all” and 5 indicates “Extremely”. Patients will be asked if and how their lifestyle habits could be dangerous for their health status and how likely they are to have caused their CVDs, from both an individual perspective and in comparison with other people of the same age and gender. Self-efficacy will be investigated by asking participants to assess their ability to adopt a healthier lifestyle on a five-point Likert scale, where 1 indicates “Not able at all” and 5 indicates “Extremely able”. Intention to change will be assessed by asking participants to evaluate the strength of their intention to change their current habits, from “I do not intend to change” to “I strongly intend to change”. Positive and negative outcome expectancies will be investigated by asking the probability of the occurrence of several events if a new healthier behavior is adopted. Both positive and negative expectancies (e.g., “My health would get better”, “My physical appearance would get better”, “Other people would appreciate my willpower”, “It would prevent further cardiovascular diseases”) and negative (“It would take a long time to became a habit”, “I would feel nervous”, “My social life would become worse”) will be measured using a five-point Likert scale, where 1 indicates “Very unlikely” and 5 indicates “Very likely”. The plan to change lifestyle habits will be investigated using a three-point Likert scale question where 1 indicates “I’ve made no plans at all” and 3 indicates “I’ve made a lot of plans”. This will evaluate if patients have made concrete plans about changing their behavior. 

#### 2.4.6. Illness Perception

The brief illness perception questionnaire (Brief-IPQ) [38] is used to measure patients’ perceptions of their disease. The Brief IPQ is a self-report scale of eight-items, five of which assess cognitive illness representations (consequences, timeline, personal control, treatment control, identity) two assess emotional representations (concern and emotions) and one item assesses illness comprehensibility. All the items are measured using a five-point Likert scale.

#### 2.4.7. Needs for Information

Patients’ information needs will be investigated with two questions evaluating the need for further information (in addition to the information already held) in six domains related to the management of hypertension or ACS [27,39]. These domains are:

- “Pharmacological Treatment”: typology of drugs, how and when to take them, possible side effects;
- “Knowledge About the Disease”: the anatomical/functional nature connected to the disease (ex. how the blood circulation system functions, what the symptoms connected to the health problem are, and what can be done to manage them);
- “Daily Activities”: information about everyday activities that can be carried out and which ones have to be modified (ex. work, free time, sexual activity);
- “Behavioral Habits”: information on lifestyle, with a focus on smoking, diet, alcohol, and physical activity;
- “Impact of the Disease”: advice on how to manage distress caused by the disease;
- “Risk and Complications”: the risks related to the disease and possible complications (e.g., the possibilities of a heart attack, how to avoid complications, who to call in case of need, etc.).

The first question asks to “Indicate how much information you would like to receive about the following topics connected to the management of your hypertension”. The answer format is on a five-point Likert scale ranging from 1 (“I want to know nothing about the topic”) to 5 (“I want to know everything about it”). The second question asks to rate the importance of the six domains assigning a value from 1 to 6 (“Now please rate the importance of the topics listed below; you must assign a value from 1 for the most important topic, to 6 for the least important one).

#### 2.4.8. Medical Adherence

Self-reported medication adherence is measured with a modified version (new items were added) of the Self-reported Measure of Medication Adherence [40] at baseline and at six-month follow-up. This adherence measure is designed to facilitate the identification of barriers to and behaviors associated with adequate adherence to chronic medications. Medication adherence scores can range from 0 to 8 with lower scores indicating lower adherence. Medical adherence has been shown to have high concordance with antihypertensive medication pharmacy fill rates in a managed care population similar to the current study population [41].

### 2.5. Intervention

The tailored print intervention will be guided by the HAPA model. This model postulates that people’s health behaviors are determined by different patterns of social-cognitive predictors [self-efficacy, outcome expectancies, and risk perception] that may emerge within two distinguished phases: (1) a goal-setting phase, with pre-intentional motivation processes that lead to a behavioral intention and (2) a goal-pursuit phase, with post-intentional volition processes that lead to the actual health behavior. In the first phase, the formation of an intention to change health behavior commences and is influenced by three factors: risk perception, outcome expectancies, and perceived self-efficacy. In the second phase, the intended behavior has to be initiated and then maintained over the longer term and even restarted in the event of setbacks. As reported in recent reviews [36,42], HAPA has been widely used in research to guide intervention to promote different health behaviors, including diet [43], alcohol consumption [44], and physical activity [45].

In the TTCYB intervention, HAPA is used to identify patients who are located either in the goal-setting phase or in the goal-pursuit phase. Then, each group receives specific messages that are tailored to the group. The basic idea is that individuals pass through different phases on their way to behavior change. Thus, interventions may be most efficient when tailored to these particular phases.

The constructs operationalized as part of the intervention include the intention to change, self-efficacy, outcome expectations, risk perception, adherence to medical treatment, need for information, and patients’ behavior. The messages developed for each phase and construct are presented below.

A library of texts and graphics files has been created to address all the variables on which the messages are customized. The messages were written by a group composed of psychology faculty members and doctoral students, who have experience in research with patients affected by cardiovascular diseases. The content of the messages has been evaluated by specialists who care for patients with chronic disease in clinical settings. Minor revisions were made based on the recommendations of these specialists. After the messages were written, reading levels were assessed and revisions were made if the level was too high. Appropriate pictures were found to enhance the quality of the brochures.

#### 2.5.1. Tailored Group

Participants randomized to the tailored group will receive tailored health material about ten days following the baseline assessment.

The tailored material consists of a nine-page full-color booklet containing a set of information the patients need to know to better manage the disease. The first part of the brochure contains a set of information related to the illness (medical information on the disease and the pharmacological treatment, information on how to manage the distress and the change in life due to the illness, information on the risks and complications of CVDs); the second part is focused on the lifestyle habits (diet, physical activity, alcohol intake, and smoking behavior) that should be modified after a CVD diagnosis.

The two parts are tailored and address different variables. The first part provided personalized information on

- Adherence to medical treatment. For example, if a patient answers “yes” to the question “Sometimes if you feel worse when you take the medicine, do you stop taking it?”, he/she will receive a message like “Pay attention, because the treatment for hypertension must not be interrupted without a physician’s approval”.
- Illness perception. For example, if a patient answers “For a very short time” to the question “How long do you think your illness will continue?”, he/she will receive a message like “Please note that acute coronary syndrome is a chronic disease; this means that it is a long-lasting condition that can be controlled but not cured and you’ll need to take pharmacological treatment for life”.

The second part will be based on

- Descriptive feedback on the behavior. For example, “Based on your answers, we determined that your daily consumption of fruits and vegetables is not healthy”. Descriptive feedback is one of the effective strategies used to tailor communication [46], to stimulate patients’ self-referential thought or otherwise focusing attention on specific behaviors related to the outcome of interest.
- HAPA constructs. In line with HAPA, health messages will be customized to the patients’ intention to change. Specific messages will be developed to address the social-cognitive predictors emerging in the different phases. Patients will be divided into five different stages of changes:
- Patients who do not intend to change their behavior (non-intenders). This group will be divided into two subgroups:
  (1) Non-intenders A—patients who do not want to change their behaviors because they have the wrong idea about the relationship between lifestyles and illness (patients who answer “I do not believe it will make my health better” or “No particular reasons” to the question “Do you intend to change your behaviors in the next months?”). For non-intenders A, the tailored messages target patients’ outcome expectancies resulting from the change in behavior, focusing on positive effects and reducing negative outcomes.
  (2) Non intenders B—patients who do not want to change due to a lack of or low level of self-efficacy (I know I will never manage to do it). For this group, the tailored messages focus on self-efficacy, to improve patients’ perception that they can change and to reduce the cognitive barriers to change.
- Patients who have not (yet) set a goal to act (preintenders), but who might consider changing their behavior. For this group, the tailoring procedure is the same as non-intenders A.
- Patients who have set a goal to change their behavior but who are not yet acting (intenders). In this case, tailored messages present some real plans to implement the change, focusing on those plans the patients declare themselves unable to foresee. The social-cognitive predictors that will be addressed are action planning, which is the subject’s ability to identify real goals to put the change into action, and coping planning, which pertains to the anticipation of barriers that might arise during the acceptance and adoption of new behavior.
- Patients who already perform the behavior in question (actors). The tailored communication focuses on the possible obstacles that patients believe that they cannot overcome to maintain the new behavior. The social-cognitive predictors that will be addressed are maintenance self-efficacy and recovery self-efficacy. The former is about the subject’s confidence that he/she can maintain a difficult behavior, while recovery self-efficacy is about the subject’s belief he/she can resume a difficult behavior after an interruption.

Table 2 presents a summary of the tailoring scheme for the development of the tailored messages related to behavioral lifestyles.

#### 2.5.2. Non-Tailored Group

The non-tailored group will complete the same questionnaire as the tailored group; participants in this group will be mailed a generic health promotion brochure promoting lifestyle changes. The generic material consists of an 8-page full-color booklet containing a generic set of information related to the illness and the lifestyle habits that should change after a diagnosis of hypertension or ACS.

#### 2.5.3. Usual Care Group

The usual care group will receive usual care from their physician, and they will complete the same assessment questionnaire as the other two groups. They will not be mailed any health promotion brochure.

### 2.6. Measurement of Outcomes

Patients will complete a self-report questionnaire six months post-randomization, and they will be asked to have a telephone interview twelve months post-randomization.

#### 2.6.1. Change in Lifestyle Habits

The primary outcome will be the change in behaviors, as measured six and 12 months after baseline. Changes in diet, physical exercise, alcohol intake, and smoking behavior will be evaluated singularly.

#### 2.6.2. Physiological Parameters

The secondary outcomes to be measured will be changes in physiological parameters related to (a) body mass index (BMI), (b) waist circumference; (c) blood pressure values, (d) the existence of diabetes mellitus, and (e) medical adherence between the baseline and the six-month follow-up.

#### 2.6.3. Patients’ Judgments about the Material

About seven days after the dispatch of the material, patients in the T and NT group will receive a telephone call in which they will be asked to judge the materials. The telephone survey will consist of dichotomous and five-point Likert scale questions. Table 3 explains the multiple questions in detail.

The same procedure (health material and telephone interview) will be conducted after the six-month follow-up.

#### 2.6.4. Potential Moderators of Intervention Efficacy

Even though there is strong evidence on the greater efficacy of tailored print materials in changing health behavior [13,14,15,16,17,18,19], there is still a lack of evidence on the moderator variables for which tailored materials work in certain conditions and for a certain population. Previous studies have called for new research to identify the reasons tailoring works in some groups and not in others [19,47] but stopped short of suggesting which variables to study. The TTCYB intervention also has the aim to understand if psychological variables related to locus of control, self-efficacy, anxiety, depression, and illness perception could moderate the efficacy of the tailored print material.

#### 2.6.5. Locus of Control

Patients’ beliefs about their disease are assessed with the Multidimensional Health Locus of Control Scale (MHLC), Form C [48], which makes it possible to detect if a patient considers health-related outcomes as a result of his/her actions, luck or chance, or the influence of others such as health professionals or family members. It is an 18-item self-report measure on a six-point scale ranging from 1 (strongly disagree) to 6 (strongly agree). It has been used in studies to make predictions about health behavior [49] and has shown good reliability and validity in a variety of chronic illness populations [48].

#### 2.6.6. Anxiety and Depression

Anxiety and depression are evaluated through the Hospital Anxiety and Depression Scale (HADS), a self-administered questionnaire specifically developed to detect states of anxiety and depression in the setting of hospital out-patient clinics [50]. It is composed of two seven-item scales (one for anxiety and one for depression), which should be used as two separate measures of emotional disturbance. All items are scored on a four-point scale from 0 to 3.

#### 2.6.7. General Self Efficacy

General self-efficacy (not specifically related to behavioral change) is assessed with the General Self-Efficacy Scale, a 10-item scale designed to assess optimistic self-beliefs to cope with a variety of difficult demands in life [51]. Possible responses are not at all true (1), hardly true (2), moderately true (3), and exactly true (4), yielding a total score between 10 and 40.

### 2.7. Sample Size Estimation

The power analysis was conducted in G*Power (G*Power, Brunsbüttel, Germany) on a repeated-measures ANOVA with within-between interaction with three measurements, three groups, with a correlation among repeated measures about 0.5, a power of 0.80, an alpha level of 0.05, and a small effect size (f = 0.10) [52]. The required sample size was found to be a minimum of 204 patients.

### 2.8. Data Collection Methods and Data Management

Each center’s personnel will be trained centrally in the study requirements, standardized measurement and the eliciting of information from study participants in a uniform reproducible manner. All data will be entered electronically at the Coordinating Center (University of Milan Bicocca). Original study forms will be entered and kept on file at the Coordinating Center. All forms, files, and datasets related to study data will be kept in locked cabinets. Access to the study data will be restricted. A password system will be utilized to control access; these passwords will be changed regularly. All reports prepared by study group members will be prepared such that no individual subject can be identified.

### 2.9. Planned Statistical Analysis

Demographic data (e.g., age, gender) will be described for the total group and the subgroups separately. Continuous variables will be denoted with means, standard deviations, and medians. Categorical variables will be denoted in numbers and percentages. Group differences in behavior and physiological (BMI and blood pressure value) changes will be tested with a repeated-measures ANOVA and logistic and linear regression as appropriate, using the baseline values of these endpoints as covariates. Regressions will also be used to test for interactions of type of material with factors such as self, efficacy, locus of control, age, gender. This strategy will allow us to test whether the intervention works better in specific groups over others. Concerning patients’ judgments of the material, a *t*-test analysis and a Cross-table with chi-square statistic will be performed. All analyses will be conducted using the Statistical Package for the Social Sciences (SPSS) version 24.0 for Windows (SPSS Inc., Chicago, IL, USA) and MPlus software Version 7.0 (Muthén & Muthén, Los Angeles, CA, USA).

### 2.10. Ethics

The project design, procedures, and informed consent form have been approved by the Ethics Committee of the University of Milan-Bicocca and will be proposed to the Ethics Committee of the hospitals in which patients will be recruited. The language used in informed consent form and during recruitment will minimize the possibility of coercion or undue influence: patients will be clearly informed that participation in the study will be voluntary and that participation choice will not affect their healthcare process.

## 3. Discussion

This report describes the rationale, design, and methods of the TTCYB study, a randomized controlled intervention that evaluates the efficacy of a tailored print intervention to improve healthy lifestyle in four behaviors (diet, physical activity, alcohol intake, and smoking behavior). This intervention targets a vulnerable and rapidly expanding segment of the population, CVD patients. The efficacy of the intervention in the tailored group compared to the non-tailored and usual care groups will be evaluated through patients’ judgments about the materials; changes in each one of the four lifestyle behaviors considered; improvements in body mass index, systolic and diastolic blood pressure; and medication adherence. If the TTCYB is efficacious, it will have implications for the design and implementation of tailored communication programs that aim at stimulating healthy behaviors to prevent worsening in patients with chronic diseases. The anticipated strengths of the study protocol include the target population, the longitudinal design, and the analysis not only of the intervention’s efficacy but also of the possible moderators of this efficacy. Besides, the intervention suggested in this protocol uses longitudinal tailoring based on models of health behavior combined in a way that has not been tested before to the best of our knowledge. The duration of the follow-up is one year, so it will be possible to investigate the long-term effects of the intervention. This project addresses major health issues and aims to contribute to the empirical and theoretical basis for developing effective primary and secondary preventive interventions. The proposed methodology can also be applied to other health problems that can benefit from lifestyle interventions. Finally, concepts from this study can potentially be extended to the primary prevention of cardiovascular diseases among high-risk groups.

## 4. Conclusions

Several studies show that substantial reductions in the CVD risk can be obtained through lifestyle changes but data regarding the long-term effects of tailored lifestyle interventions among cardiovascular patients are still lacking.

This article describes a novel protocol, the “Time to Change Your Behavior” (TTCYB) study protocol, a theory-based, tailored print message intervention to improve cardiovascular patients’ lifestyles. The project has the potential to affect preventive health programs, given that TTCYB could be a new intervention capable of preventing clinical worsening and relapses in chronic patients. More research will be needed to determine how to integrate the protocol into routine healthcare processes.

## Figures and Tables

**Table 1:** Overview of measures over the different time points of the study.

	Baseline	Time 2—Six Months after Baseline	Time 3—12 Months after Baseline
Lifestyle behaviors	x	x	x
HAPA constructs	x	x	
Illness perception	x	x	
Needs for Information	x	x	
Medical adherence	x	x	
Locus of control	x	x	
Anxiety and depression	x	x	
General Self Efficacy	x	x	
Clinical evaluation	x	x	

**Table 2:** Tailoring scheme for the development of messages related to the lifestyle.

Patients’ Intention to Change Stage	Social-Cognitive Predictor	Example of a Tailored Message
Non intender a	Outcome Expectancy	*“You reported that in changing your diet you are afraid about the difficulty to buy the right products. Don’t be afraid of this, because, especially in recent years, there is growing attention towards a healthy diet. The greater transparency that has been reached in the communication properties of the food will certainly help you to choose the best groceries. You have to always remember to BRING ON YOUR TABLE ONLY FOOD THAT ALLOWS YOU TO FEEL GOOD”.*
Non intender b	Self-Efficacy	*“When you answered the questionnaire, you declared that don’t want to change your diet because you thought you would not be able to do so. Trying to change your nutrition is not so difficult as it sounds. Try to make one change at a time; it is not necessary to change everything at once. Start, for example, by some very simple things, maybe planning the day in which to start your diet and mark it on the calendar. You can also start by searching for a nutrition specialist, who could give you important advice”.*
Preintender	Outcome Expectancy	
Intender	Action Planning	*Planning how to start changing your diet is the first step to actualize the intention to change. From the answers at the questionnaire, it emerged that you have no plan on how to manage the situation in which is difficult to maintain the change (e.g., the lunch at work). Try to organize your lunch by preparing it at home in order to avoid buying unhealthy stuff.*
Actor	Maintenance and Recovery Self-Efficacy	*Do not worry if your partner and/or your family would not change their eating habits with you. It is not necessary that everyone in the family changes habits. Probably when they will see the positive outcomes that you will achieve with your diet, they will ask you for advice and suggestion to eat like you!*

**Table 3:** Telephone survey to collect patients’ judgments after the delivery of the material.

Questions on a Five-Point Likert Scale (1 = not at All; 2 = Few; 3 = on Average; 4 = Sufficiently; 5 = Very	Dichotomous Question (Yes/No)
The material was attractive?	Have you read the material?
The material was easy to understand?	Have you stored the material?
The material encouraged your reflection?	Have you shown the material to others?
The material increased your knowledge about hypertension?	Would you read the material again in the future?
The material was personalized on your specific situation?	Was the information in the material new?
The material contained information pertinent to your specific situation?	Do you think it would be useful to receive this kind of material again in the future?
The material was complete?	
Would you make behavioral changes based on the material (specific for diet, physical activity, alcohol intake, and smoking behavior)?	

## Trial Registration

The trial was retrospectively registered with the ISRCTN registry (Trial ID: ISRCTN13415993; date of registration: 8 April 2019).
